# Cell-permeable organic fluorescent probes for live-cell long-term super-resolution imaging reveal lysosome-mitochondrion interactions

**DOI:** 10.1038/s41467-017-01503-6

**Published:** 2017-11-03

**Authors:** Yubing Han, Meihua Li, Fengwu Qiu, Meng Zhang, Yu-Hui Zhang

**Affiliations:** 1 Britton Chance Center for Biomedical Photonics, Wuhan National Laboratory for Optoelectronics-Huazhong University of Science and Technology, Wuhan, Hubei 430074 China; 2 MoE Key Laboratory for Biomedical Photonics, Collaborative Innovation Center for Biomedical Engineering, School of Engineering Sciences, Huazhong University of Science and Technology, Wuhan, Hubei 430074 China

## Abstract

Characterizing the long-term nanometer-scale interactions between lysosomes and mitochondria in live cells is essential for understanding their functions but remains challenging due to limitations of the existing fluorescent probes. Here, we develop cell-permeable organic fluorescent probes for lysosomes with excellent specificity and high photostability. We also use an existing Atto 647N dye with high brightness and excellent photostability to achieve specific labeling of mitochondria in live cells. Using these probes, we obtain dual-color structured illumination microscopy (SIM) images of dynamic physical lysosome-mitochondrion interactions in live cells at an ~90-nm resolution over a long time course of ~13 min. We successfully record the consecutive dynamic processes of lysosomal fusion and fission, as well as four types of physical lysosome-mitochondrion interactions by super-resolution imaging. Our probes provide an avenue for understanding the functions and the dynamic interplay of lysosomes and mitochondria in live cells.

## Abstract (web summary)

Studying interactions between lysosomes and mitochondria in living cells is difficult due to the limitations of existing probes. Here, the authors develop new cell-permeable fluorescent probes to image the dynamics of lysosomes and their physical interactions with mitochondria using super-resolution microscopy.

## Introduction

Extensive research has indicated that intracellular organelles are integrated into cellular networks and collaborate on various cellular tasks rather than acting as isolated entities^1,2^. Specialized membrane contact sites are formed between organelles, providing distinct spatial regions for executing and regulating their functions^1^. Lysosomes and mitochondria are crucial dynamic organelles that participate extensively in many important cellular processes. Their dysfunction has been implicated in diverse diseases such as neurodegenerative disorders, cancer, and cardiovascular diseases^3,4^. However, few studies of the dynamic physical interactions (referring to the interactions involving direct physical contacts rather than signaling pathways) between lysosomes and mitochondria have been reported^2,5,6^, and it remains unknown whether there are membrane contacts between them in mammalian cells, despite the likely importance of their interplay in apoptosis, autophagy, and cellular aging^7^. Conventional fluorescence microscopy permits noninvasive imaging of lysosomes and mitochondria in live cells, but it suffers from an optical diffraction-limited spatial resolution that is insufficient for resolving the membrane boundaries between lysosomes and mitochondria^8^. Electron microscopy has been exploited to identify sites of physical contact between organelles, but it is incapable of capturing dynamic processes in live cells^8,9^. The recently developed structured illumination microscopy (SIM)^10^ displays substantially improved spatiotemporal resolution and provides a powerful approach for examining the dynamics of subcellular structures in live cells^11,12^. However, the limitations of existing fluorescent lysosomal and mitochondrial probes, such as photobleaching and a nonspecific background^13,14^, hinder the characterization of dynamic physical interactions between lysosomes and mitochondria in live cells.

In this study, we first develop a series of cell-permeable organic fluorescent probes for lysosomes with different colors that exhibit high specificity and excellent photostability. We then explore the possibility of an existing Atto 647N dye with high fluorescence intensity and excellent photostability as a live-cell mitochondrial marker. Using our lysosomal probes and Atto 647N, we obtain dual-color SIM images of dynamic physical lysosome-mitochondrion interactions in live cells at a ~90-nm resolution over a long time course of ~13 min.

## Results

### Design and synthesis of the cell-permeable lysosomal probes

We designed and synthesized a series of cell-permeable organic fluorescent probes for lysosomes that consist of a cell-penetrating peptide (rR)_3_R_2_^15^, a recognition unit (an epoxysuccinyl scaffold that selectively forms covalent bonds with cysteine cathepsins^16,17^), and a fluorescent dye (Supplementary Fig. 1). Alexa Fluor 647, Atto 565, and Atto 488 were used to construct the probes (termed Lysosome-647, Lysosome-565, and Lysosome-488, respectively) due to their excellent optical properties (Supplementary Table 1). Remarkably, using our synthetic strategy (Supplementary Notes 1–2), other commercially available dyes with an N-hydroxysuccinimidyl (NHS) group, regardless of their cell permeability, were easily incorporated into cell-permeable lysosomal probes, thus significantly expanding the number of lysosomal probes available for live-cell super-resolution multi-color imaging.

### Characterization of the lysosomal probes in live cells

**Fig. 1:**
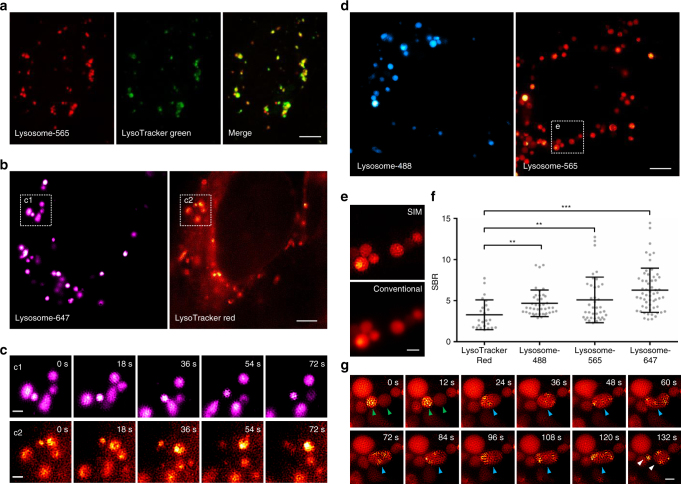
Characterization of the lysosomal probes in live cells. **a** A co-localization study employing LysoTracker Green as the standard lysosomal marker. Live U2OS cells were simultaneously stained with Lysosome-565 (red) and LysoTracker Green (green, 50 nM) for 30 min and imaged by confocal microscopy. **b** Dual-color SIM images of lysosomes in live U2OS cells stained with both Lysosome-647 (magenta) and LysoTracker Red (red, 50 nM). **c** Enlarged time-lapse images from the boxed regions shown in **b**. **d** SIM images of live U2OS cells after incubation with Lysosome-488 (blue) or Lysosome-565 (red). **e** Enlarged super-resolution SIM (upper panel) and diffraction-limited (lower panel) images from the boxed region shown in **d**. **f** Average signal (lysosomes) to background (cytosol) ratios (SBR) of LysoTracker Red (SBR from n = 24 areas), Lysosome-488 (SBR from *n* = 41 areas), Lysosome-565 (SBR from *n* = 40 areas), and Lysosome-647 (SBR from *n* = 58 areas) in three SIM images (mean ± s.d.; ^**^ *P* < 0.01, ^***^ *P* < 0.0001, two-tailed t-test; statistics were performed using SPSS 19.0 software package (IBM Co.)). **g** Time-lapse images illustrating the process of lysosomal fission-fusion. Each SIM frame was acquired over 270 ms (i.e., a raw data exposure time of 30 ms). For the time-lapse images, consecutive SIM frames spaced at 6-s intervals were obtained; representative images of consecutive SIM frames are displayed (more frames are shown in Supplementary Movie 1). Scale bars, **a** 5 μm, **b**, **d** 4 μm, and **c**, **e**, **g** 1 μm

We first investigated the cell permeability of our lysosomal probes. Confocal images (Supplementary Fig. 2a–c) reveal that, after incubation with the live cells for 30 min, all of Lysosome-647, Lysosome-565, and Lysosome-488 entered cells efficiently and exhibited a punctate vesicular pattern. Lysosome-565 co-localized well with both LysoTracker Green (the most popular commercially available organic fluorescent acidotropic probe for lysosomes, Pearson’s coefficient: 0.776, Fig. 1a) and GFP-LAMP1 (Green Fluorescent Protein–Lysosome Associated Membrane Protein 1, Pearson’s coefficient: 0.811, Supplementary Fig. 2d), indicating the high specificity of Lysosome-565. We then evaluated the cytotoxicity of the lysosomal probes in live cells by using 3-(4,5-dimethylthiazol-2-yl)-5-(3-carboxymethoxyphenyl)-2-(4-sulfophenyl)-2H-tetrazolium (MTS) assay. The results indicate that after incubation with Lysosome-488, Lysosome-565, or Lysosome-647 for 30 min, all of the cell viabilities were higher than 85%, suggesting that these lysosomal probes exhibit low levels of cytotoxicity (Supplementary Fig. 3). Cathepsin B and cathepsin H have been shown to be the top 2 most abundant cathepsins targeted by the epoxysuccinyl scaffold^17^, therefore, we also determined the activities of cathepsin B and cathepsin H in live cells after treatment with Lysosome-565 or Lysosome-647 by using Cathepsin B/H Activity Assay Kits. Our results reveal that after incubation with Lysosome-565 or Lysosome-647 for 30 min, the activities of both cathepsin B and cathepsin H in live cells were maintained >77% of untreated control samples, implying that these lysosomal probes have limited impacts on the activities of cathepsins (Supplementary Fig. 4).

On the basis of the excellent cell permeability, high specificity, and low cytotoxicity of our lysosomal probes, we investigated the utility of these probes in a live-cell SIM imaging. The open-source SIMcheck software was used to assess data quality of SIM images, and SIM images with obvious full-field artifacts were excluded^18^. After staining with Lysosome-647, Lysosome-565, or Lysosome-488, the shapes of lysosomes were clearly demarcated in live-cell SIM images with low-fluorescence background (Fig. 1b–f). Moreover, after incubation with different concentrations of Lysosome-565, no obvious difference of SBR (signal-to-background ratio) was observed (Supplementary Fig. 5a). To avoid a potential cytotoxicity of the probes at a high concentration, we performed our following experiments with solutions of the lysosomal probes prepared by diluting 43 µL of the stock solution with PBS to a final volume of 100 µL. In contrast, at 50 or 100 nM (recommended working concentrations according to the manufacturer’s instructions), LysoTracker Red showed an uneven background in the perinuclear region, making the SIM images too blurry to recognize the shapes of lysosomes (Fig. 1b, c, f and Supplementary Fig. 5b and 6a, b, e). At higher concentrations (500 nM or 1 µM), LysoTracker Red suffered from both high background and nonspecific mitochondrial labeling (Supplementary Fig. 5b and 6c, d).

With Lysosome-565, we successfully recorded the consecutive dynamics of lysosomal fusion and fission by SIM imaging (Fig. 1g and Supplementary Movie 1). We observed two lysosomes (identified with green arrowheads in Fig. 1g) with distinct fluorescent intensities fusing to form a hybrid organelle (blue arrowheads) that continued to change shape. After ~100 s, the hybrid organelle finally split into two (white arrowheads) or three lysosomes (identified with green arrowheads in Supplementary Fig. 7 and Supplementary Movie 2) with distinct sizes. Some hexagonal structures inside lysosomes were observed in Fig. 1g and the analysis by using SIMcheck indicates that these hexagonal structures were mainly caused by fast motion of the contents inside lysosomes (Supplementary Fig. 8). We mainly focused on the changes of the membrane boundaries of lysosomes rather than the distribution of contents inside lysosomes, therefore, the hexagonal structures have little effect on the observation of the process. The lysosomal fission and fusion events in live cells have been described by conventional microscopy^19,20^. To the best of our knowledge, this is the first time that the consecutive dynamic processes of lysosomal fission and fusion in live cells have been observed by super-resolution imaging. We have directly visualized the process of lysosomal fusion, as well as the subsequent re-formation of lysosomes, therefore providing new insights into the dynamic behavior of lysosomes in vivo.

### Characterization of Atto 647N for mitochondria marking

**Fig. 2:**
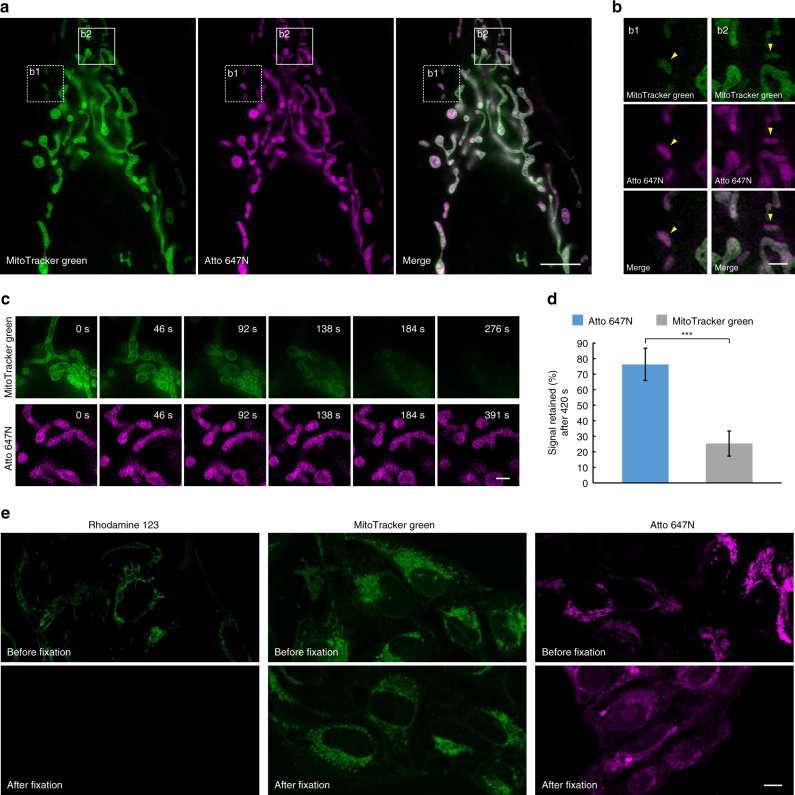
Characterization of Atto 647N in live cells. **a** A co-localization study employing MitoTracker Green as the standard mitochondrial marker. Live U2OS cells were simultaneously stained with MitoTracker Green (green, 400 nM) and Atto 647N (magenta, 15 µM) for 30 min and imaged by SIM. **b** Enlargements of the boxed regions in **a**. **c** Time-lapse SIM images of live U2OS cells stained with MitoTracker Green (green, 400 nM) or Atto 647N (magenta, 15 μM). **d** The percentage of retained fluorescence intensity of Atto 647N and MitoTracker Green after 420 s of time-lapse SIM imaging (from *n* = 10 areas, mean ± s.d.; ^***^ *P* < 0.0001, two-tailed t-test; statistics were performed using SPSS 19.0 software package (IBM Co.)). **e** Confocal images of U2OS cells stained with Rhodamine 123 (2 µM), MitoTracker Green (400 nM), or Atto 647N (15 µM) before and after cell fixation. Each SIM frame was acquired over 270 ms (i.e., a raw data exposure time of 30 ms). For the time-lapse images, consecutive SIM frames spaced at ~ 1.15-s intervals were obtained; representative images of consecutive SIM frames are displayed. Scale bars, **a** 5 μm, **b**, **c** 1 μm, and **e** 10 μm

Atto 647N is a recently developed fluorescent dye and the optimal candidate for SIM imaging among commonly used dyes, given its high fluorescence intensity and excellent photostability (Supplementary Table 1). Although Atto 647N has been reported to have a high affinity for mitochondria in immunofluorescence staining^21^, whether it can be used as a mitochondrial marker in live cells remains unknown. After incubation with live U2OS cells for 30 min, Atto 647N NHS ester (referring to as Atto 647N in this paper) successfully stained mitochondria with a high average SBR (Supplementary Figs. 9 and 10) and co-localized well with MitoTracker Green (a widely used commercially available mitochondrial marker^22,23^, Pearson’s coefficient: 0.885, Fig. 2a). No obvious difference of SBR was observed after incubation with different concentrations of Atto 647N (6 or 15 µM, Supplementary Fig. 10) and 15 µM of Atto 647N was employed in subsequent SIM experiments. It is worth noting that besides numerous rod-like, network-like, and granular mitochondria, mitochondria with smaller sizes and unusual shapes (such as puncta) were also observed, which were stained by both Atto 647N and MitoTracker Green (identified with yellow arrowheads in Fig. 2b). Remarkably, no obvious photobleaching or mitochondrial swelling was observed within 7 min of applying Atto 647N (excitation power: 0.71 W cm^−2^; Fig. 2c, d). In contrast, a relatively high illumination intensity (excitation power: 6.43 W cm^−2^) was required to detect MitoTracker Green due to its low brightness, which caused obvious mitochondrial swelling and photobleaching within 2 min (Fig. 2c, d). After 7 min, the fluorescence intensity of MitoTracker Green was reduced to ~25% of the initial values while ~75% of the initial fluorescence intensity of Atto 647N remained (Fig. 2d).

Previous studies have demonstrated that the antifading agent Trolox (6-hydroxy-2,5,7,8-tetramethylchroman-2-carboxylic acid) could reduce photobleaching^24–26^ and inhibit mitochondrial swelling^27^, so we tried to add Trolox in our SIM experiments. The results reveal that after the addition of Trolox, no obvious mitochondrial swelling was observed within 8 min while obvious mitochondrial swelling occurred at 3 min without Trolox (Supplementary Fig. 11). Therefore, Trolox was used in subsequent experiments. The cytotoxicity of Atto 647N was also evaluated by MTS assay. The result indicates that after incubation with Atto 647N for 30 min, the cell viabilities were higher than 86%, suggesting the low cytotoxicity of Atto 647N (Supplementary Fig. 3).

Next, we tried to investigate the mechanism behind the specificity of Atto 647N toward mitochondria. The commonly used fluorescent mitochondrial marker can be divided into two main groups based on their chemical structure. One group includes some cationic dyes (e.g., Rhodamine 123 and tetramethylrhodamine) which selectively accumulate in mitochondria depending on negative mitochondrial membrane potential^28^. After cell fixation, these dyes could not label mitochondria any more due to loss of membrane potential^29^. The other group consists of cationic dyes containing active groups (e.g., MitoTracker probes containing a mildly thiol-reactive chloromethyl moiety) which can form covalent bonds with proteins on mitochondrial membrane^29^. They maintain their ability to label mitochondria after cell fixation. We incubated live U2OS cells with Rhodamine 123, MitoTracker Green, or Atto 647N for 30 min, and then fixed the cells with 4% paraformaldehyde before washing and imaging. The results (Fig. 2e) reveal that after cell fixation, the fluorescence of Rhodamine 123 almost disappeared within the cells. In contrast, cell fixation had little effect on the mitochondrial labeling pattern of Atto 647N and MitoTracker Green. Considering that it is also a cationic carbopyronine dye and contains an active group (a NHS moiety), Atto 647N seemed to label mitochondria due to its affinity toward mitochondrial membrane negative potential difference and binding to mitochondrial membrane proteins, similar to MitoTracker probes^29^.

### Long-term SIM imaging of lysosome–mitochondrion interactions

Previous reports have shown that the cross-talk between lysosomes and mitochondria is involved in important signaling pathways and cellular processes, such as vesicle transport and mitophagy^6,30^. However, few studies have examined the dynamic physical interactions between mitochondria and lysosomes in live cells due to the limitations of the existing lysosomal and mitochondrial probes, leading to an incomplete understanding of the complex interplay between mitochondria and lysosomes^2,5,6^. Therefore, we tried to explore the utility of our probes in live-cell dual-color SIM experiments to reveal the lysosome-mitochondrion interactions.

**Fig. 3:**
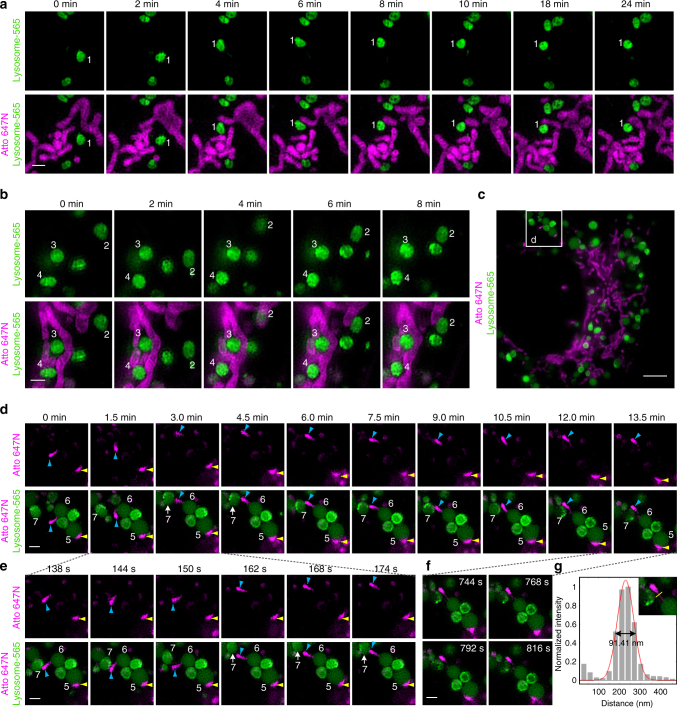
Dual-color SIM images of lysosomes and mitochondria in live cells. **a**, **b**, **d**, **e**, **f** Representative time-lapse SIM images reveal different types of dynamic physical interactions between lysosomes and mitochondria in live U2OS cells stained with Lysosome-565 (green) and Atto 647N (magenta) (more frames are shown in Supplementary Movies 3–5). **c** A SIM image of a whole live U2OS cell. **g** Cross-sectional profile of the tubular structure of the mitochondrion in **f** at a full-width at the half-maximum (FWHM) of 91 nm. Each SIM frame was acquired over 270 ms (i.e., a raw data exposure time of 30 ms). For the time-lapse images, the time interval between each dual-color SIM image was set to 6 s. Scale bars, **a**, **b**, **d**, **e**, **f** 1 μm, and **c** 4 μm

We first determined the photostability of Lysosome-565 and Atto 647N in live-cell dual-color SIM experiments. The results indicate even after 13.5 min, >90% of the initial fluorescence intensity of both Atto 647N (excitation power: 0.71 W cm^−2^) and Lysosome-565 (excitation power: 5.71 W cm^−2^) remained (Supplementary Fig. 12). On the basis of the high specificity (Figs. 1a and 2a) and excellent photostability of Lysosome-565 and Atto 647N (Supplementary Fig. 12), we then performed long-term dual-color SIM imaging of lysosomes and mitochondria in live cells (Fig. 3, and Supplementary Movies 3–5). Remarkably, by applying our probes in SIM imaging, we were able to record the dynamic process of physical lysosome-mitochondrion interactions in live cells with a spatial resolution of ~90 nm (Fig. 3g).

We identified four types of dynamic physical interactions between lysosomes and mitochondria. First, some lysosomes (e.g., lysosomes No. 1 in Fig. 3a and No. 2 in Fig. 3b and Supplementary Movies 3 and 4) initially moved rapidly and then came into contact with and adhered to a mitochondrion. Second, some lysosomes (e.g., lysosomes No. 3 and 4 in Fig. 3b and Supplementary Movie 4) were surrounded and trapped within the mitochondrial network and were limited in their motility along the mitochondrial network from beginning to end. Third, mitochondria that were smaller than lysosomes (e.g., mitochondrion identified with yellow arrowheads in Fig. 3d–e and Supplementary Movie 5) remained bound to a lysosome (No. 5) and moved with it for the duration of the study. Remarkably, we also recorded a process of dynamic transfer of a mitochondrion between two lysosomes (Fig. 3d–f and Supplementary Movie 5). The mitochondrion (identified with blue arrowheads) initially wandered in the cytoplasm with one end adherent to a lysosome (No. 6); then, the mitochondrion attached to another lysosome (No. 7) at its other end. After stretching between the two lysosomes for ~10 min, the mitochondrion ultimately detached from the first lysosome (No. 6) and transferred to the other lysosome (No. 7). It is worth noting that the entire course of this transfer was as long as 13 min (Fig. 3d) and that some mitochondrial and lysosomal movements occurred on a timescale of a few seconds (Fig. 3e). Moreover, during the transfer process, the mitochondrion formed a thin and tubular intermediate structure with a full width at half maximum (FWHM) of ~90 nm (Fig. 3f–g). Thus, we provide visual evidence of dynamic physical lysosome-mitochondrion interactions at a high spatial resolution and over a long time course of ~13 min. To the best of our knowledge, this is the first time that dynamic physical interactions between lysosomes and mitochondria have been characterized by super-resolution imaging, implying the existence of contact sites between them in mammalian cells^5,6^.

### Application of Lysosome-565 and Atto 647N to mitophagy

Mitophagy is a process that eliminates damaged mitochondria by sequestrating them with autophagosomes and subsequent degrading them in autolysosomes. Mitophagy maintains a healthy pool of mitochondria and therefore plays an important role in keeping cells healthy^31,32^. Defects in mitophagy have been associated with neurodegenerative diseases and cardiac disorders^33–35^. Fluorescence microscopy has been widely used to profile the co-localization and dynamics of mitochondria with lysosomes during mitophagy, however, usually blurry images were obtained due to the limitations of existing fluorescent probes and the limited spatial resolution of conventional microscopy in previous reports^36^. Therefore, we tried to utilize our probes in the studies of mitophagy by SIM imaging.

**Fig. 4:**
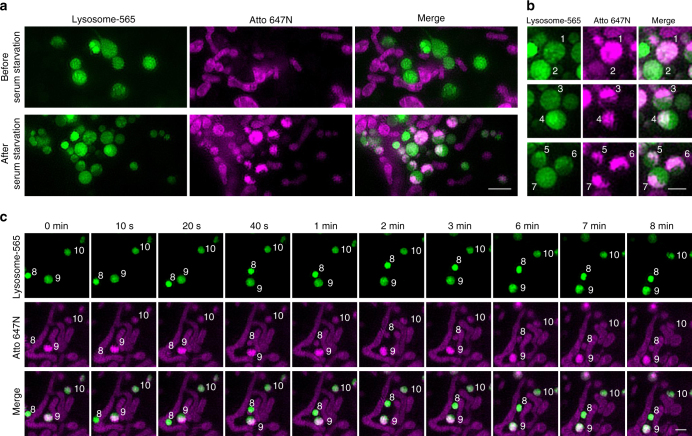
Determination of co-localization of mitochondria with lysosomes during mitophagy. **a** Dual-color SIM images of lysosomes (green) and mitochondria (magenta) before and after serum starvation in live U2OS cells. Live U2OS cells stained with Lysosome-565 (green) and Atto 647N (magenta) were incubated with culture medium without fetal bovine serum (FBS) for 4 h before imaging. **b** Representative mitochondria within lysosomes with diverse shapes. **c** Time-lapse SIM images reveal dynamics of the autolysosomes after serum starvation (more frames are shown in Supplementary Movie 6). Each SIM frame was acquired over 270 ms (i.e., a raw data exposure time of 30 ms). For the time-lapse images, the time interval between each dual-color SIM image was set to 10 s; representative images of consecutive SIM frames are displayed. Scale bars, **a** 2 μm, and **b**, **c** 1 μm

Several methods have been reported to induce mitophagy, such as the addition of an uncoupler carbonyl cyanide m-chlorophenylhydrazine (CCCP)^37^, starvation treatment^38^, and laser-induced photodamage^39^. We used serum starvation to induce mitophagy in our experiments. After stained with Lysosome-565 and Atto 647N, live U2OS cells were serum-starved for 4 h and then imaged by dual-color SIM. The results indicate that both Lysosome-565 and Atto 647N maintained their staining during mitophagy (Fig. 4). Remarkably, the SIM images not only accurately determined the co-localization of autolysosomes and mitochondria but also clearly demarcated the shapes of both autolysosomes and the mitochondria within them. Some mitochondria fully filled the autolysosomes (lysosomes No. 1 and 2 in Fig. 4b) and some occupied a part of the autolysosomes (lysosomes No. 3–7 in Fig. 4b). Moreover, the time-lapse SIM images indicate that the autolysosomes containing mitochondria (lysosomes No. 8–10 in Fig. 4c and Supplementary Movie 6) exhibited similar behaviors to normal lysosomes (lysosomes No. 1–4 in Fig. 3a, b). Taken together, these results indicate that Lysosome-565 and Atto 647N can be used for dual-color SIM imaging in the studies of mitophagy, demonstrating their potential for use in a wide array of applications.

In conclusion, we developed a series of cell-permeable organic fluorescent probes for lysosomes with high specificity and excellent photostability. We also successfully used an existing Atto 647N dye with high fluorescence intensity and excellent photostability to label mitochondria with high specificity in live cells. By applying our probes in SIM imaging, we successfully recorded the consecutive dynamic processes of lysosomal fusion and fission as well as physical lysosome-mitochondrion interactions in live cells by super-resolution imaging. We also successfully applied our probes in the studies of mitophagy. The results here provide an avenue for understanding the functions and the dynamic interplay of lysosomes and mitochondria in live cells. Studies using our probes to explore the interactions between lysosomes, mitochondria, and other subcellular structures, as well as the molecular biological mechanisms behind them are currently underway.

## Methods

### Synthesis of lysosomal probes

The organic probes for lysosomes were constructed from two parts. One part (referred to as the peptide part, detailed structure shown in Supplementary Fig. 1) contains the recognition unit (epoxysuccinyl scaffold), the cell-penetrating peptide (rR)_3_R_2_, and a short peptide GKGKGK, in which lysines offer free active amino groups available to conjugate with commercially available dyes via an N-hydroxysuccinimidyl (NHS) group. The other part is a commercially available fluorescent dye containing an N-hydroxysuccinimidyl (NHS) group. The two parts were linked via covalent bonds to form an entire lysosomal probe (see Supplementary Note 1). The peptide part was prepared by solid-phase peptide synthesis (Supplementary Note 2) and was purified by preparative high-performance liquid chromatography (HPLC) to a purity of >95%, and its mass was confirmed by electrospray ionization mass spectrometry (EI-MS) (Supplementary Fig. 1b, c). Before conjugation to dyes, the peptide part was dissolved in bicarbonate buffer (0.1 M, pH 8.3) at a concentration of 1 mM and stored at 4 °C.

The commercial available dyes Alexa Fluor 647 (Thermo Fisher Scientific, Inc.), Atto 488 (Sigma-Aldrich Co., LLC), and Atto 565 (Sigma-Aldrich Co., LLC) containing a N-hydroxysuccinimidyl (NHS) moiety were dissolved with anhydrous dimethylformamide (DMF; Sigma-Aldrich Co., LLC), divided into small aliquots in multiple tubes (40 μg of Alexa Fluor 647, 21 μg of Atto 565, or 30 μg of Atto 488 in each tube), evaporated to dryness in a vacuum centrifuge, and stored at −20 °C.

For the conjugation, an aliquot of each dye was dissolved with 10–20 µL of anhydrous dimethyl sulphoxide (DMSO; Sigma-Aldrich Co., LLC) or DMF, added to 14 µL of the peptide part solution, and mixed thoroughly. The mixture was allowed to react at room temperature overnight with constant shaking. Then the mixture was purified using Pierce C18 Spin columns (Thermo Fisher Scientific, Inc.) according to the manufacturer’s instructions. After purification, the supernatant was evaporated to dryness in a vacuum centrifuge and the residue was dissolved in 200 µL of Phosphate Buffered Saline (PBS, pH 7.4; Thermo Fisher Scientific, Inc.) to generate a stock solution of the lysosomal probe.

### Cell culture

Human osteosarcoma cell line (U2OS, Cat. Number: CX0318) cells were purchased from Boster Biological Technology Co., Ltd., Wuhan, China, and cultured in McCoy’s 5A medium (Thermo Fisher Scientific, Inc.). All media were supplemented with 10% (v/v) fetal bovine serum (FBS; Thermo Fisher Scientific, Inc.), and the cultures were maintained at 37 °C in a humidified 5% CO_2_ environment. For starvation treatment, live U2OS cells were incubated in McCoy’s 5 A medium without FBS for 4 h before imaging.

### Live-cell labeling

For lysosomal staining, U2OS cells were seeded in Nunc Glass Bottom Dishes (Φ 12 mm, Thermo Fisher Scientific, Inc.) at a density of 1.5–2.0 × 10^4^ per well in growth medium (150 µL). After an overnight incubation, the cells were washed three times with PBS. Solutions of the indicated lysosomal probes at different concentrations were prepared by diluting different volumes (i.e., 14–71 µL) of the stock solution with PBS to a final volume of 100 µL. After adding a solution of the indicated probe diluted in PBS, the cells were incubated in a 5% CO_2_ atmosphere at 37 °C for 30 min. Then, the supernatant was discarded, and the cells were post-incubated with growth medium in a 5% CO_2_ atmosphere at 37 °C for 4 h prior to SIM imaging. For dual staining, other probes were added to the dishes after the 4 h incubation with growth medium.

For mitochondrial labeling, an aliquot of Atto 647N (25 μg, Sigma-Aldrich Co., LLC) was dissolved in 10 µL of DMSO and was diluted with PBS to a total volume of 100 µL (3–15 µM). The cells were then incubated with the probe solution in a 5% CO_2_ atmosphere at 37 °C for 30 min; afterwards, the supernatant was discarded.

For commercially available probes, the cells were incubated with LysoTracker Green (50 nM, 100 µL; Thermo Fisher Scientific, Inc.), LysoTracker Red (50–1000 nM, 100 µL; Thermo Fisher Scientific, Inc.) or MitoTracker Green (400 nM, 100 µL; Thermo Fisher Scientific, Inc.) in a 5% CO_2_ atmosphere at 37 °C for 30 min; afterwards, the supernatant was discarded.

Before imaging, a solution of Trypan blue (100 µL, 1 mg·mL^−1^; Sigma-Aldrich Co., LLC) in PBS was added to exclude the dead cells and quench the extracellular fluorescence from the probes bound to either the cell membrane or the dish surface^40^. After 1 min, Trypan blue was removed, and the cells were washed twice gently with PBS and immersed in phenol red-free DMEM (Thermo Fisher Scientific, Inc.) with 1 mM (±)−6-hydroxy-2,5,7,8-tetramethylchromane-2-carboxylic acid (Trolox; Sigma-Aldrich Co., LLC) prior to optical imaging.

### MTS assay

The cytotoxicity of the probes for lysosomes and mitochondria on U2OS cells was tested using a MTS assay. Solutions of the lysosomal probes were prepared by diluting 43 µL of a stock solution with PBS to a final volume of 100 µL. U2OS cells (4 × 10^3^ cells per well) were seeded into a 96-well plate and cultured in growth medium for 24 h. The cells were incubated with the solution of the probes (Lysosome-488, Lysosome-565, Lysosome-647, or Atto 647N (15 µM)) in PBS in a 5% CO_2_ atmosphere at 37 °C for 30 min. Then the supernatant was replaced by 100 µL of growth medium, and 20 µL of CellTiter 96 AQueousOne Solution Reagent (Promega Co.) was added into each well. The cells were incubated for 3 h at 37 °C in a 5% CO_2_ atmosphere. The absorbance was recorded at 492 nm using a TECAN GENios Plus ELISA reader (Tecan, Inc.). The cell viabilities were expressed as the percentage of the A492 of the probe-treated cells to the untreated controls, and all of the measurements were performed in triplicate.

### Determination of cathepsin activity

Cathepsin B/H Activity Assay Kits (Abnova Co., Ltd.) were used to determine cathepsin B/H activity in live cells. Live U2OS cells were incubated with solutions of Lysosome-565, Lysosome-647, or untreated at 37 °C for 30 min, respectively. Solutions of the indicated lysosomal probes were prepared by diluting 43 µL of the stock solution with PBS (pH 7.4) to a final volume of 100 µL. The cells were post-incubated with growth medium for 4 h to reduce background fluorescence. Then the cells were dissociated, and 2 × 10^5^ of the cells were collected in 1.5-mL Eppendorf tubes by centrifugation and lysed in 10 μL of chilled Cell Lysis Buffer for 10 min. Then the tubes were centrifuged 15,700 ×g for 5 min and the clear lysate was transferred into new tubes. 3 μL of the clear lysate, 50 μL of Cell Lysis Buffer, 50 μL of Reaction Buffer, and 2 μL of Substrate Ac-RR-AFC/R-AFC were added into 96 wells. For determining the background, 50 μL of Cell Lysis Buffer, 50 μL of Reaction Buffer, and 2 μL of Inhibitor were added into 96 wells. The solutions were mixed well and incubated at 37 °C for 1 h. Results were analyzed using a PerkinElmer Envision fluorescence plate reader (Excitation/Emission = 400/505 nm; PerkinElmer, Inc.), as described in the kit instructions. The cathepsin activities were expressed as the percentage of the relative fluorescence units of the probe-treated cells to the untreated controls, and all of the measurements were performed in triplicate.

### Confocal laser scanning microscopy

The images were obtained using a LSM-710 confocal laser scanning microscope (Carl Zeiss, Inc.) equipped with a 63×/1.49 numerical aperture oil-immersion objective lens and were analyzed with ZEN 2012 (Carl Zeiss, Inc.) and ImageJ software (National Institutes of Health). All fluorescence images were analyzed and the background subtracted with ImageJ software. Pearson’s coefficient^41^ was quantified using the Colocalisation Analysis plugin for ImageJ.

### SIM imaging

Super-resolution images were acquired on a N-SIM microscope (Nikon Instruments, Inc.) equipped with an Apochromat 100 × /1.49 numerical aperture oil-immersion objective lens and solid-state lasers (488 nm, rated power ≥ 200 mW; 561 nm, rated power ≥ 150 mW; 647nm, rated power ≥ 100 mW). Images were captured with an electron-multiplying charge coupled device (EMCCD) camera (Andor; 512 × 512 px, 14 bit) with a gain value of 100.

Raw SIM images (containing nine images: three phases and three angles) were obtained and reconstructed with Nikon Elements software (Nikon Instruments, Inc.). The exposure time was set to 30 ms for each raw data capture (each single-color SIM frame was acquired over 270 ms in total). SIM frames were deliberately spaced at 1-s, 6-s, 10-s or 1-min intervals according to the purpose of each experiment. SIM images were analyzed with Nikon Elements and ImageJ software, and tested by using the ImageJ plugin SIMcheck^18^ to assess the image quality. Detailed information regarding the imaging conditions used for SIM imaging is summarized in Supplementary Table 2.

### Data availability

The authors declare that all data supporting the findings of this study are available within the article and its Supplementary Information files or from the corresponding authors on reasonable request.

## Electronic supplementary material

Supplementary Information

Description of Additional Supplementary Files

Supplementary Movie 1

Supplementary Movie 2

Supplementary Movie 3

Supplementary Movie 4

Supplementary Movie 5

Supplementary Movie 6
