# Supplementary material for: Cell-permeable organic fluorescent probes for live-cell long-term super-resolution imaging reveal lysosome-mitochondrion interactions
Source: Nat Commun. 2017 Nov 3;8:1307. doi: 10.1038/s41467-017-01503-6 (PMC5670236; doi:10.1038/s41467-017-01503-6)
Supplement: Supplementary file 1 — Supplementary Information [file 41467_2017_1503_MOESM1_ESM.pdf]

a

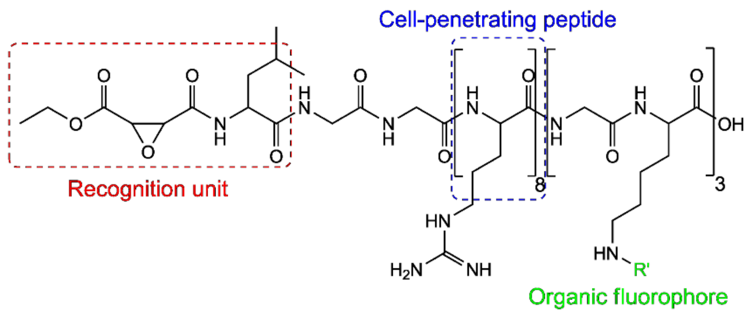

b

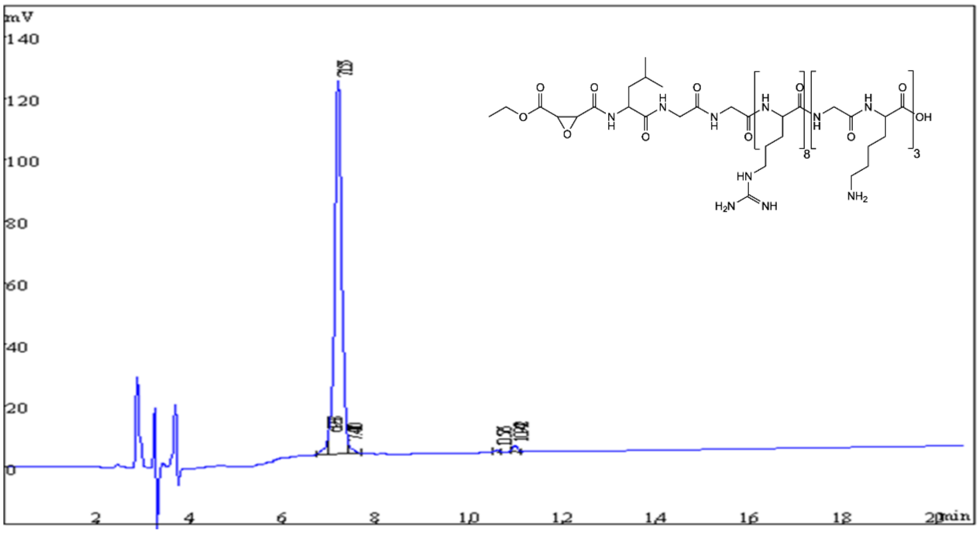

Rank	Time (min)	Conc. (%)	Area
1	6.933	1.793	24040
2	7.155	96.22	1290292
3	7.4	0.905	12135
4	10.576	0.2841	3810
5	10.942	0.7935	10640
Total		100	1340917

c

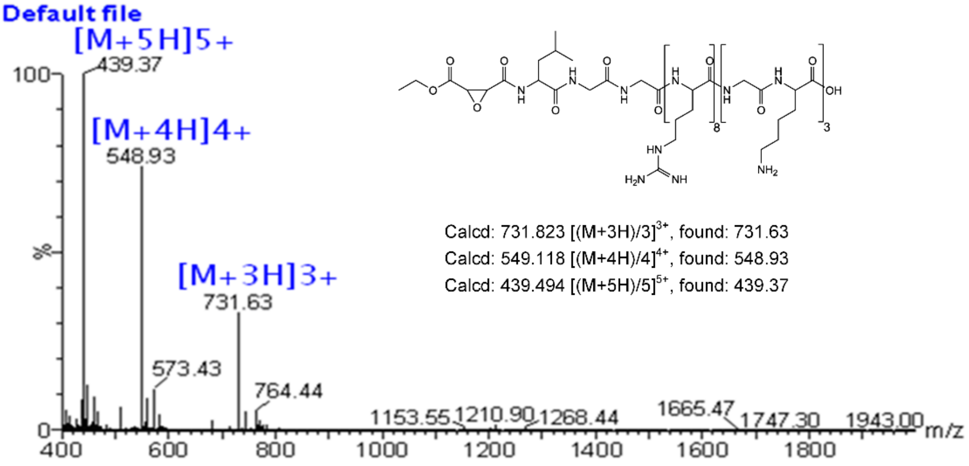

Supplementary Figure 1. (**a**) Structures of the lysosomal probes. (**b**) HPLC analysis of the peptide part of the lysosomal probes. (**c**) ESI mass spectrum analysis of the peptide part of the lysosomal probes.

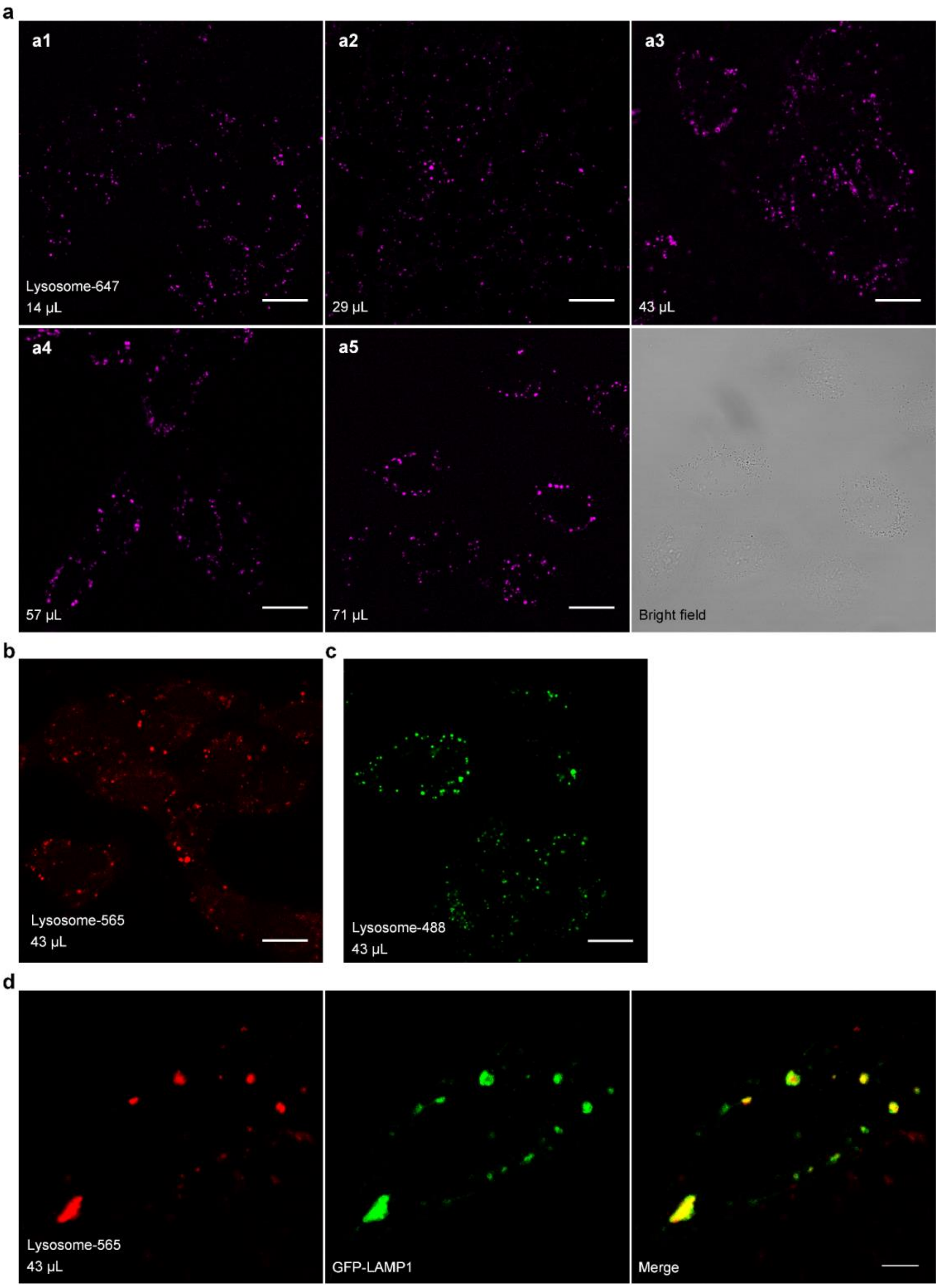

Supplementary Figure 2. Confocal microscopy images of live U2OS cells after incubation with (**a**) Lysosome-647, (**b**) Lysosome-565 or (**c**) Lysosome-488 for 30 min before imaging. Solutions of Lysosome-647 at different concentrations were prepared by diluting different volumes [i.e., (**a1**) 14 µL, (**a2**) 29 µL, (**a3**) 43, (**a4**) 57 µL, or (**a5**) 71 µL] of the stock solution with PBS to a final volume of 100 µL. Solutions of Lysosome-565 and Lysosome-488 were prepared by diluting 43 µL of the stock solution with PBS to a final volume of 100 µL. (**d**) A co-localization study employing GFP-LAMP1 as the standard lysosomal marker. Live U2OS cells transiently transfected by GFP-LAMP1 (green) were stained with Lysosome-565 (red, 43 µL) for 30 min, post-incubated for 4 h and imaged by confocal microscope. Scale bars, (**a-c**) 20 µm, and (**d**) 5 µm.

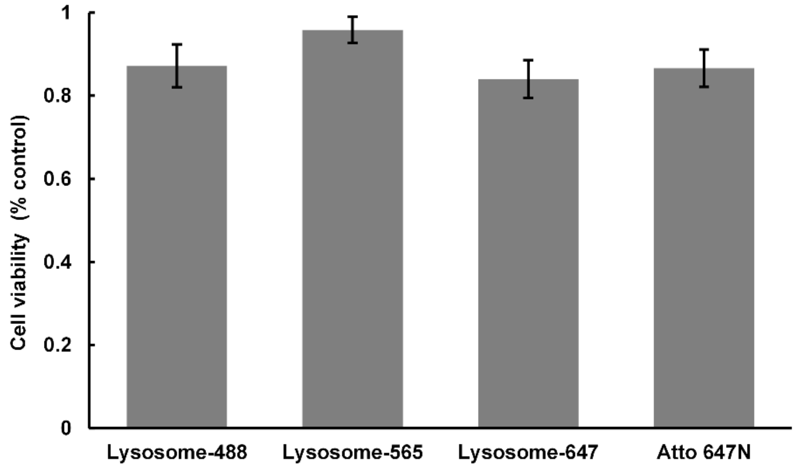

Supplementary Figure 3. U2OS cell viabilities after a 30-min incubation with a solution of the probes (Lysosome-488, Lysosome-565, Lysosome-647, or Atto 647N) in PBS at 37 ℃. Solutions of the lysosomal probes were prepared by diluting 43 μL of a stock solution with PBS to a final volume of 100 μL. The concentration of Atto 647N is 15 μM. Error bars represent the standard deviations of triplicate experiments.

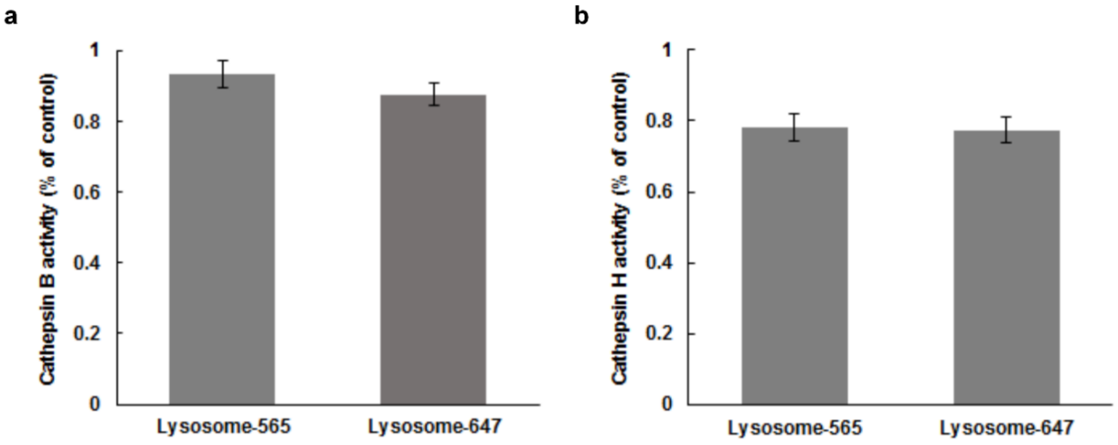

Supplementary Figure 4. Activities of cathepsin B (**a**) and cathepsin H (**b**) in live U2OS cells after incubation with Lysosome-565 or Lysosome-647 for 30 min. Solutions of the lysosomal probes were prepared by diluting 43 μL of a stock solution with PBS to a final volume of 100 μL. Error bars represent the standard deviations of triplicate experiments.

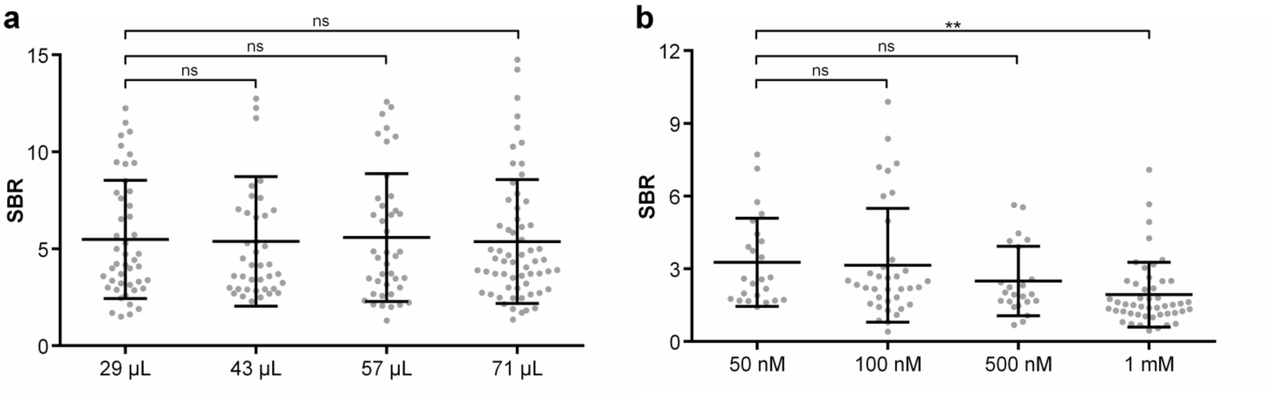

Supplementary Figure 5. Average signal (lysosomes) to background (cytosol) ratios (SBR) of (**a**) Lysosome-565 (SBR from n = 46, 40, 41, 61 areas, respectively) and (**b**) LysoTracker Red (SBR from n = 24, 36, 23, 48 areas, respectively) at different concentrations (mean ± s.d.; ns = $P > 0.05$; **$P < 0.01$, two-tailed t-test; statistics were performed using SPSS 19.0 software package (IBM Co.)). Solutions of Lysosome-565 at different concentrations were prepared by diluting different volumes [i.e., 29 μL, 43 μL, 57 μL, or 71 μL] of the stock solution with PBS to a final volume of 100 μL.

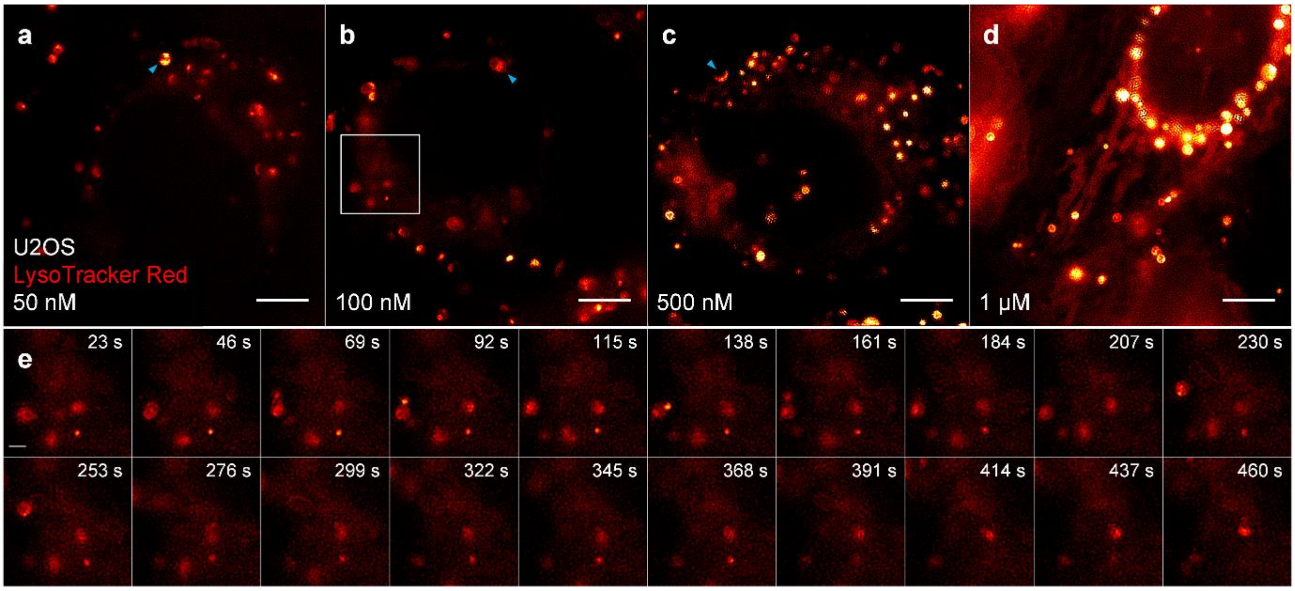

Supplementary Figure 6. SIM images of live U2OS cells stained with different concentrations of LysoTracker Red. Live U2OS cells were incubated with (**a**) 50 nM, (**b**) 100 nM, (**c**) 500 nM, or (**d**) 1 μM LysoTracker Red for 30 min before imaging. (**e**) Enlarged time-lapse images from the boxed region shown in **b**, showing a dynamic background of LysoTracker Red. Each SIM frame was acquired over 270 ms (i.e., a raw data exposure time of 30 ms). For time-lapse images, consecutive SIM frames spaced at 1-s intervals were obtained, and representative images of consecutive SIM frames are displayed. Scale bars, (**a-d**) 5 μm, and (**e**) 1 μm.

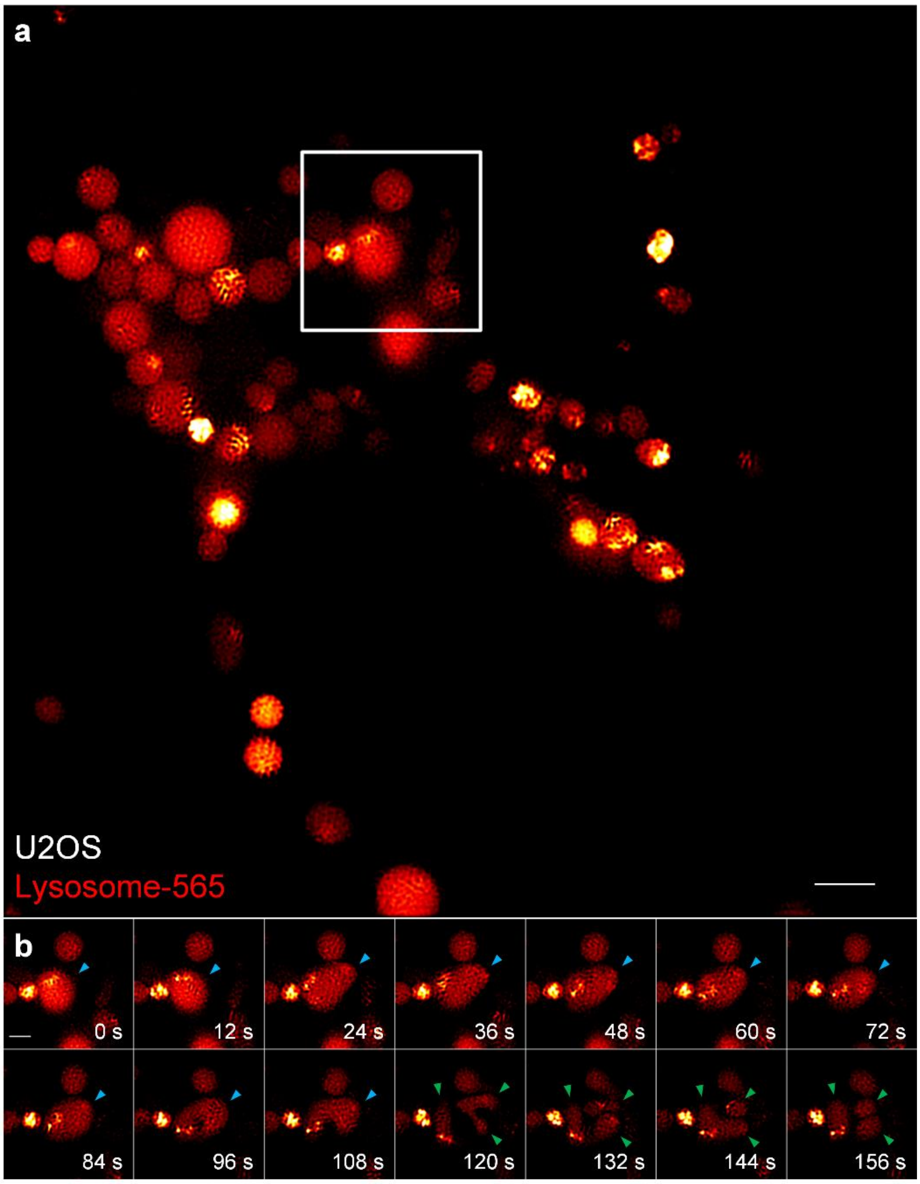

Supplementary Figure 7. SIM images of lysosomes exhibiting fission dynamics. (**a**) Live U2OS cells were stained with 43 μL of Lysosome-565 for 30 min before imaging. (**b**) Time-lapse images from the boxed region shown in **a**. Each SIM frame was acquired over 270 ms (i.e., a raw data exposure time of 30 ms). For time-lapse images, consecutive SIM frames spaced at 6-s intervals were obtained, and representative images of the consecutive SIM frames are displayed (more frames are shown in Supplementary Video 2). Scale bars, (**a**) 2 μm, and (**b**) 1 μm.

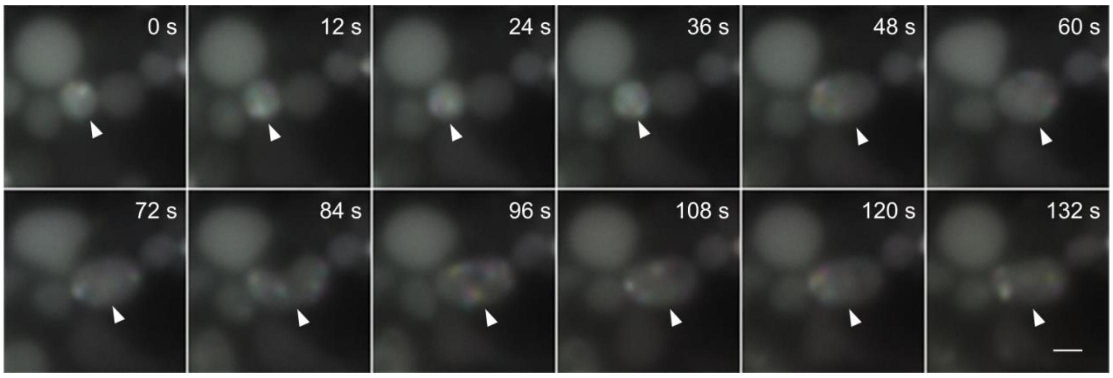

Supplementary Figure 8. Motion and illumination variation test of raw data of Figure 1g by using SIMcheck. The round shapes of non-white areas (cyan, magenta, or yellow, identified with white arrowheads) suggest that the differences between angles arising from fast particle motion lead to the hexagonal structures. Scale bars, 1 μm.

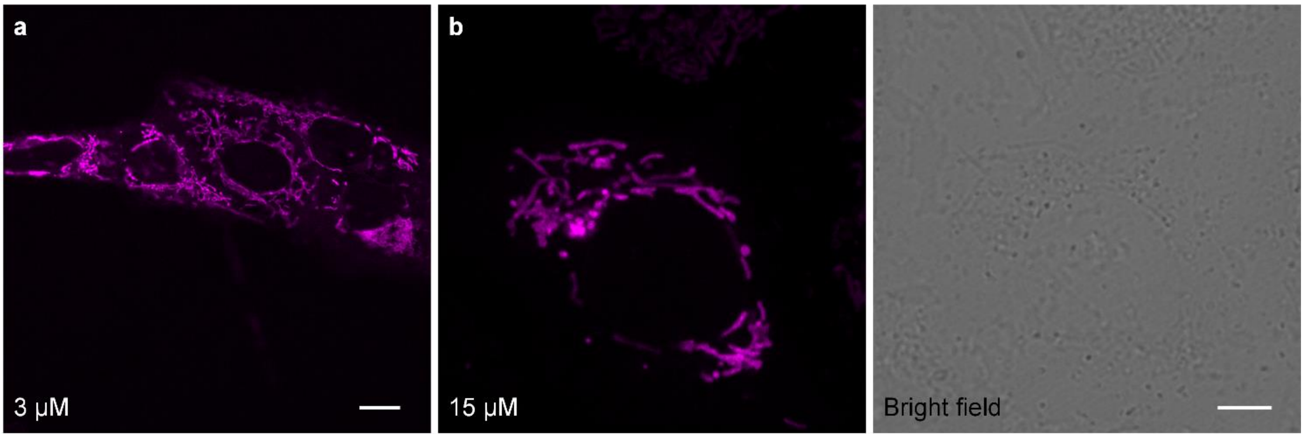

Supplementary Figure 9. Confocal microscopy images of live U2OS cells after incubation with (a) 3 μM or (b) 15 μM Atto 647N for 30 min before imaging. Scale bars, (**a**) 10 μm, and (**b**) 5 μm.

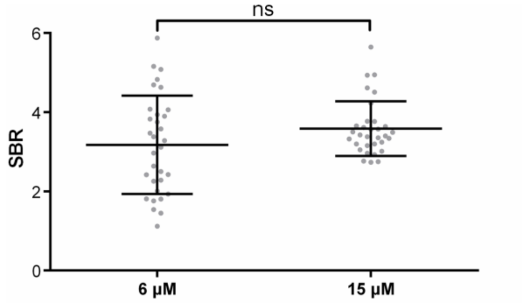

Supplementary Figure 10. Average signal (mitochondria) to background (cytosol) ratios (SBR) of Atto 647N at 6 μM (SBR from n = 32 areas) in three SIM images, or 15 μM (SBR from n = 32 areas) in two SIM images (mean ± s.d.; ns = $P > 0.05$; two-tailed t-test; statistics were performed using SPSS 19.0 software package (IBM Co.)).

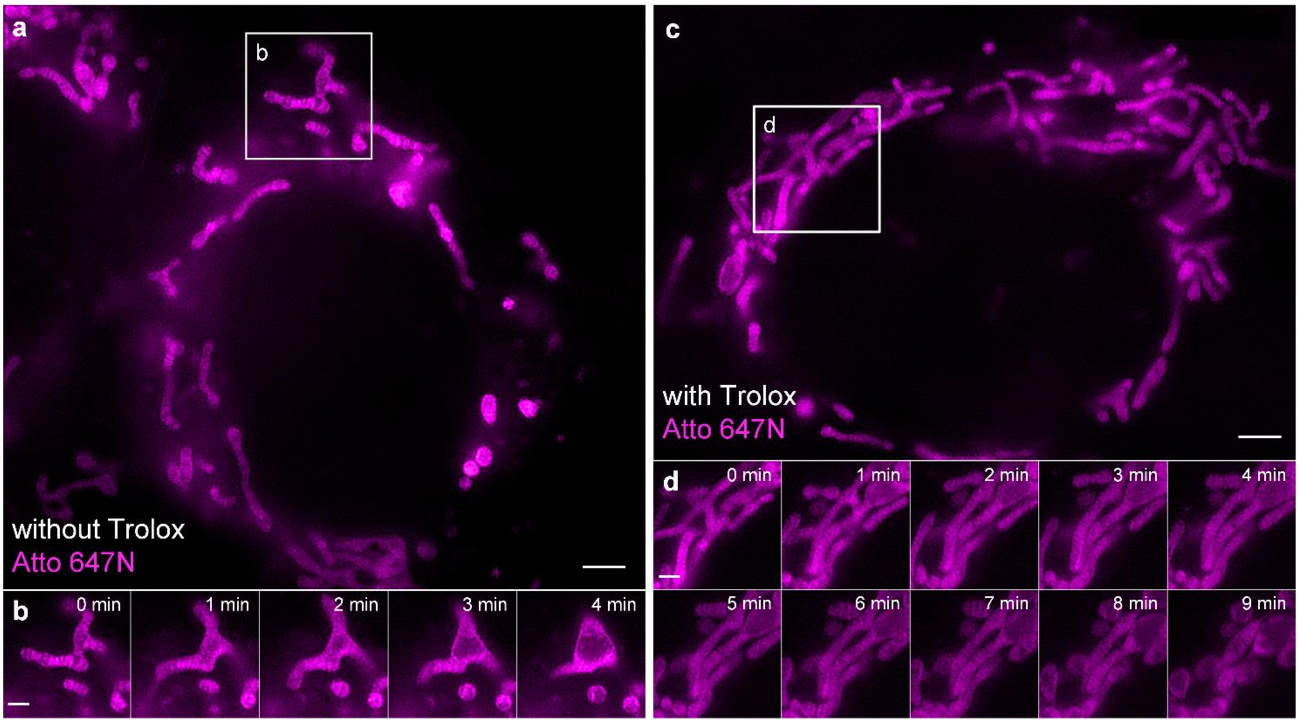

Supplementary Figure 11. The effects of Trolox on mitochondrial swelling during SIM experiments**.** SIM images of live U2OS cells stained with Atto 647N (15 μM) (a) without or (c) with the addition of Trolox (1 mM). (b, d) Time-lapse images from the boxed regions shown in **a** and **c**, respectively. For time-lapse images, consecutive SIM frames spaced at 1-s intervals were obtained, and representative images of consecutive SIM frames are displayed. Scale bars, (**a**, **c**) 5 μm, and (**b**, **d**) 1 μm.

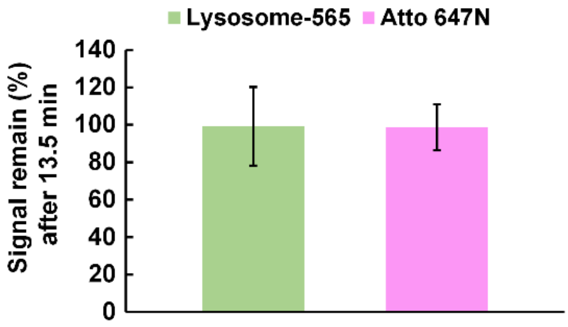

Supplementary Figure 12. The percentage of retained fluorescence intensity of Lysosome-565 (from n = 22 lysosomes) and Atto 647N (from n = 13 areas) after 13.5 min of time-lapse dual-color SIM imaging (mean ± s.d.). Each SIM frame was acquired over 270 ms (i.e., a raw data exposure time of 30 ms). For the time-lapse images, the time interval between each dual-color SIM image was set to 6 s.

Supplementary Table 1. Summary of the optical properties of frequently used fluorescent dyes or proteins.

Dye	Excitation maximum (nm)[a]	Emission maximum (nm)[a]	Extinction (m^{-1} cm^{-1})[b]	Quantum yield[c]	Lifetime (ns)
**Blue-absorbing**					
Atto 488	501	523	90,000	0.8	4.1
Alexa 488	495	519	71,000	0.92	4.1
Fluorescein	494	518	70,000	0.79	4
FITC	494	506	70,000	0.8	4.1
BODIPY FL	505	513	80,000	-	5.7
EGFP	489	508	55,000	0.6	-
EYFP	514	527	84,000	0.61	-
**Yellow-absorbing**					
Cy3	550	570	136,000	0.15	0.3
Cy3.5	581	596	150,000	0.15	0.5
Cy3B	558	572	130,000	0.67	2.8
Alexa 568	578	603	91,300	0.69	3.6
Atto 565	564	590	120,000	0.92	4
Alexa 555	555	565	150,000	0.1	3.6
Atto 550	554	576	120,000	0.8	0.3
BODIPY TR	588	617	68,000	-	5.4
DsRed	558	583	72,500	0.68	-
**Red-absorbing**					
Alexa 647	650	665	239,000	0.33	1
Atto 647	645	669	120,000	0.2	2.4
Atto 647N	644	669	150,000	0.65	3.5
Cy5	649	670	250,000	0.28	1
Cy5.5	675	695	250,000	0.28	1
SiR	645	661	100,000	0.39	-
**NIR-absorbing**					
DyLight 755	755	776	220,000	-	-
Cy7	747	776	200,000	0.28	-
Alexa Fluor 750	749	775	240,000	0.12	0.7
Atto 740	740	764	120,000	0.1	-

[a]Excitation and emission peak wavelengths of dye spectra. [b]Extinction coefficients reported by the dye manufacturers. [c]Quantum yields from either the dye manufacturer when reported or from the McNamara 2007 fluorophore data tables. -, values not available from the dye manufacturer or McNamara data tables.

Supplementary Table 2. Imaging conditions for SIM experiments.

	Label	Dosage	Excitation λ (nm)	Illumination intensity (W/cm^2)	Exposure time per raw image (ms)	Cycle time (Acquisition + resting time) (sec)	Time points
Fig. 1b-c	LysoTracker Red	50 nM	561	5.71	30	6	95
	Lysosome-647	43 µL	647	1.43	30		
Fig. 1d(left panel)	Lysosome-488	43 µL	488	2.14	30	6	99
Fig. 1d(right panel)-e	Lysosome-565	43 µL	561	5.71	30	1.15	over 300
Fig. 1g	Lysosome-565	43 µL	561	15.00	30	6	24
Fig. 2a-b	MitoTracker Green	400 nM	488	6.43	30	6	86
	Atto 647N	15 µM	647	1.43	30		
Fig. 2c(upper panel)	MitoTracker Green	400 nM	488	6.43	30	1.15	354
Fig. 2c(lower panel)	Atto 647N	15 µM	647	0.71	30	1.15	354
Fig. 3a	Lysosome-565	43 µL	561	3.21	30	60	31
	Atto 647N	15 µM	647	0.36	30		
Fig. 3b	Lysosome-565	43 µL	561	5.71	30	6	244
	Atto 647N	15 µM	647	0.71	30		
Fig. 3c-f	Lysosome-565	43 µL	561	5.71	30	6	137
	Atto 647N	15 µM	647	0.71	30		
Fig. 4a(upper panel),b	Lysosome-565	43 µL	561	3.21	30	10	10
	Atto 647N	15 µM	647	0.71	30		
Fig. 4a(lower panel)	Lysosome-565	43 µL	561	11.07	30	6	62
	Atto 647N	15 µM	647	0.71	30		
Fig. 4c-d	Lysosome-565	43 µL	561	3.21	30	10	61
	Atto 647N	15 µM	647	0.71	30		
Supplementary Fig. 6	LysoTracker Red	50-1000 nM	561	5.71	30	1.15	over 300
Supplementary Fig. 7 and 8	Lysosome-565	43 µL	561	15.00	30	6	28
Supplementary Fig. 11a-b	Atto 647N	15 µL	647	1.43	30	1.15	283
Supplementary Fig. 11c-d	Atto 647N	15 µL	647	1.43	30	1.15	602

Supplementary Note 1. Design of the lysosomal probes.

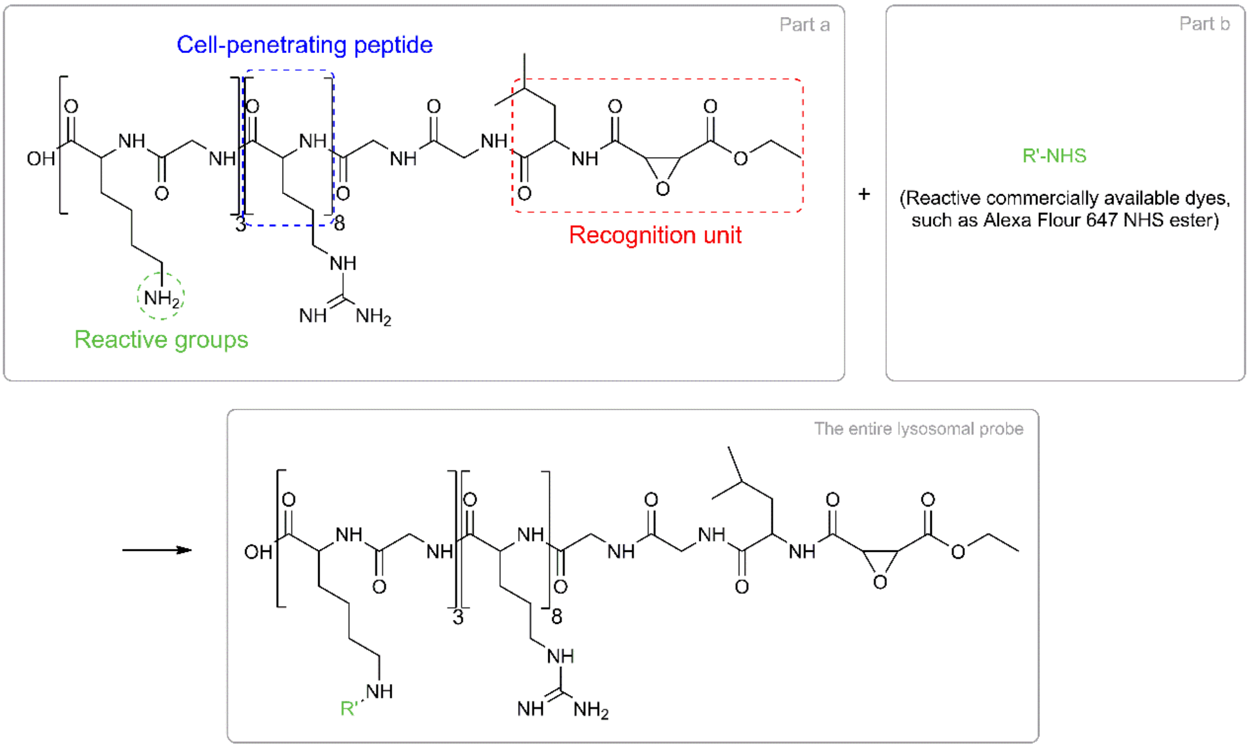

Supplementary Note 2. Synthesis of the peptide part of the lysosomal probes.

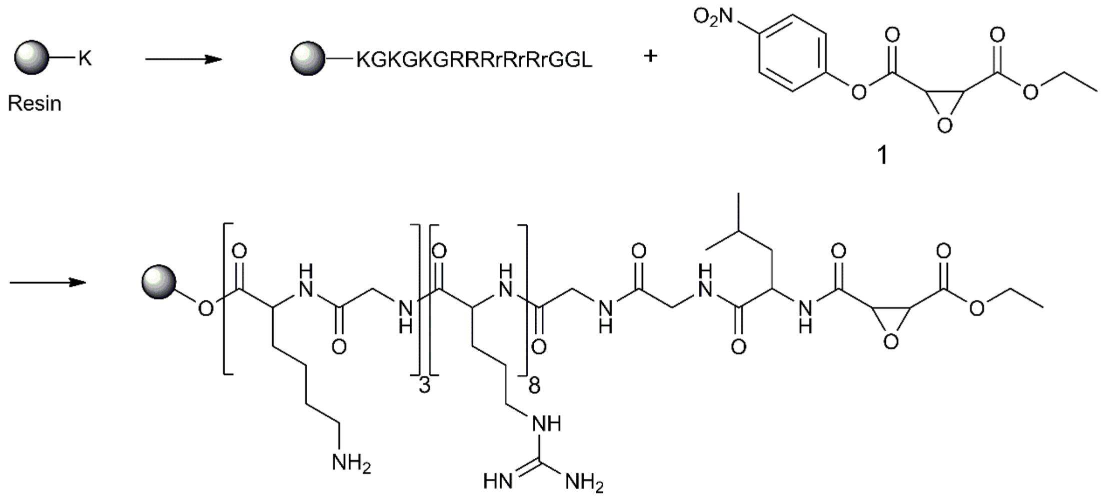

Compound 1 was synthesized as previously reported[1], and the peptide part of the lysosomal probes was synthesized using 9-fluorenylmethoxycarbonyl (Fmoc) solid-phase peptide synthesis on 2-chlorotrityl chloride resin with standard deprotection and coupling procedures[2-3]. The final product was purified by preparative HPLC to reach a purity > 95%, and the appropriate mass was confirmed by EI-MS spectrometry (Supplementary Fig. 1).

Supplementary References

1. Chehade, K. A. H. *et al.* An improved preparation of the activity-based probe JPM-OEt and in situ applications. *Synthesis* **2**, 240-244 (2005).

2. Fan, F. *et al*. Labeling lysosomes and tracking lysosome-dependent apoptosis with a cell-permeable activity-based probe. *Bioconjug. Chem.* **23**, 1309-1317 (2012).

3. Fan, F. *et al*. Protein profiling of active cysteine cathepsins in living cells using an activity-based probe containing a cell-penetrating peptide. *J. Proteome Res.* **11**, 5763-5772 (2012).
