# Supplementary material for: Cell-permeable organic fluorescent probes for live-cell long-term super-resolution imaging reveal lysosome-mitochondrion interactions
Source: Nat Commun. 2017 Nov 3;8:1307. doi: 10.1038/s41467-017-01503-6 (PMC5670236; doi:10.1038/s41467-017-01503-6)
Supplement: Supplementary file 2 — Description of Additional Supplementary Files [file 41467_2017_1503_MOESM2_ESM.pdf]

Description of Additional Supplementary Files

File Name: Supplementary Movie 1
Description: A series of time-lapse super-resolution images of the process of lysosomal fusion and fission in live U2OS cells stained with Lysosome-565 at time intervals of 6 s.

File Name: Supplementary Movie 2
Description: A series of time-lapse super-resolution images of one hybrid organelle splitting into three lysosomes in live U2OS cells stained with Lysosome-565 at time intervals of 6 s.

File Name: Supplementary Movie 3
Description: A series of time-lapse super-resolution images of lysosomes slowed down by mitochondria in live U2OS cells stained with Lysosome-565 (green) and Atto 647N (magenta) at time intervals of 1 min.

File Name: Supplementary Movie 4
Description: A series of time-lapse super-resolution images of lysosomes trapped or slowed down by mitochondria in live U2OS cells stained with Lysosome-565 (green) and Atto 647N (magenta) at time intervals of 6 s.

File Name: Supplementary Movie 5
Description: A series of time-lapse super-resolution images of the dynamic process of mitochondria that were either bound to or transferred between lysosomes in live U2OS cells stained with Lysosome-565 (green) and Atto 647N (magenta) at time intervals of 6 s.

File Name: Supplementary Movie 6
Description: A series of time-lapse super-resolution images of the dynamics of the autolysosomes after serum starvation in live U2OS cells stained with Lysosome-565 (green) and Atto 647N (magenta) at time intervals of 10 s.
